# Social Responsibility and Misleading Advertising of Health Products on the Radio. The Opinion of the Professionals

**DOI:** 10.3390/ijerph18136912

**Published:** 2021-06-28

**Authors:** María Teresa García-Nieto, Juan Enrique Gonzálvez-Vallés, Mónica Viñarás-Abad

**Affiliations:** 1 Department of Theories and Analysis of Communication, Faculty of Information Sciences, Universidad Complutense de Madrid, 28040 Madrid, Spain; nieto@ccinf.ucm.es; 2 Department of Applied Communication Sciences, Faculty of Information Sciences, Universidad Complutense de Madrid, 28040 Madrid, Spain; mvinaras@ucm.es

**Keywords:** misleading advertising, social responsibility, advertising professionals, communication and health

## Abstract

This research studies the opinion of advertising professionals in agencies, on the responsibility in relation to misleading advertising of health-related products, on the medium of radio. Through a closed survey of these professionals with different types of response, dichotomous, multiple choice and Likert scale, relevant results were obtained regarding compliance and application of the law and social responsibility linked to an advertising that directly affect health. The results show that only 10% of them know the legislation, although almost 90% of those surveyed consider it necessary to have legislative knowledge, and for only half of these, is it important. A large majority assure that the health sector should be one of the most protected sectors in the advertising world and, it should be noted, that the vast majority of the professionals surveyed view the legal restrictions on advertising in the health sector as positive. There is no unanimity as to who is responsible for the message, agency or advertiser. For its part, radio is presented as one of the most serious media and less prone to misleading advertising. To conclude, it can be stated that the professionals of the agencies do not perceive the existence of misleading advertising in the health sector, neither do they consider radio as one of the media where this deception can most occur. However, they coincide in stating that the health sector is one of the most dangerous if the damage that advertising deception can cause to consumers is considered.

## 1. Introduction

Misleading advertising is a serious detriment to consumers and, therefore, to one of the groups of interest of the advertising agencies and the advertiser with whom it is related. In the case of advertising for health products, where audiences are more vulnerable, this fact is more reprehensible. Advertisers are aware that today brands, and therefore companies, have to combine their sales objectives with a strategic vision of the organization based on ethics, transparency and CSR [1]. Nowadays, advertising has a twofold function: its role as a commercial activity and, on the other hand, it has a social dimension by directly influencing contemporary ways of life. Advertising represents one of the most regulated activities in Spain, but from a social point of view its management is more complicated and the consumer is not always protected [2].

Misleading advertising can pose a risk to citizens and consumers. Therefore, it is subject to different laws and regulations that ensure a truthful and ethical message. However, the fact that the message depends on both the advertiser and different professionals within the advertising agency, can lead to a situation where control of the rules is lost. 

For this reason, it has been considered of great interest to learn the opinion of advertising professionals regarding this situation, in order to explore whether they feel responsible and how this is managed. For its part, the radio has a large audience, with an intimate and personal tone, which justifies the interest in its study. The results will allow us to reflect on the responsibility of all the agents involved.

The main objective of this work is to understand the perception and opinion that advertising communication professionals have about misleading advertising and their liability in relation to it. Specifically, the work focuses on misleading advertising in relation to health-related products in the radio medium.

The work is structured starting from a state of the art, after having raised a brief introduction on the relevance of the topic and the objectives. Then, the methodology for presenting the relevant results is explained. The last part collects the conclusions and the discussion separately.

### 1.1. Misleading Advertising—Disloyal and Illicit

The European regulations on misleading advertising are in Directive 2006/114/EC [3], on misleading and comparative advertising. In Article 2 of the Directive, misleading advertising is considered to be “any advertising that, in any way, including its presentation, misleads or may mislead the people it addresses or affects and that, due to its misleading nature, may affect their economic behaviour or, for these reasons, harms or is capable of harming a competitor “. In Spain, misleading advertising is considered an act of unfair competition, as set out in article 5 of the text of Organic Law 3/1991 of January 10 on Unfair Competition Practices (LCD) [4]. 

Therefore, misleading advertising is essentially characterized by inducing error or deception in the possible acceptor of a contract, in such a way that the principle of good contractual faith is violated, which must prevail in every legal relationship [5]. Despite this, in advanced societies organized around the logic of mass consumption, advertisers sometimes use unfair techniques to increase or maintain their market share [6]. Therefore, in all developed countries this type of advertising is considered a crime [7]. This advertising disloyalty affects fair play and hurts competitors and the economy of the customer [8].

The advertising sector is one of the most regulated, but due to its social impact, self-regulation has acquired a fundamental role [9,10]. Self-regulation has become an indispensable way to defend consumer rights [11]. Associations such as Autocontrol [12] in Spain seek to ensure responsible advertising: loyal, truthful, honest and legal. Meanwhile, consumer associations warn of misleading and disloyal messages in relation to the nature and ownership of goods or services, their availability and after-sales services [13]. Despite this, different sectors are identified -some with a serious effect on health- where the practice of this type of advertising is a reality today [14]. 

Research on unfair advertising has been carried out from different approaches. Most research is accomplished from the perspective of legal regulations and codes of conduct, based on precepts of the FDA (Food and Drug Administration) [15,16,17]. Some studies reflect on the persuasive effects of consumer deception. Deception comes in many forms, from outright lies, to the amount and sufficiency of information, the degree of truthfulness, clarity, relevance and intent [18,19]. A study by Ukaegbu [7] reveals that some people do not find it easy to identify whether an advert is misleading. Consumers should be aware that their buying behaviour may be the result of deception by advertising methods. They must know how companies present and advertise their products or services to avoid deception [20,21]. Education constitutes a determining variable of the level of influence of this type of advertising [22]. 

The arrival of the internet and new technologies poses a challenge for misleading advertising. The most recent studies show the use of these recommendation techniques as illicit or misleading advertising, which requires a legal regulatory framework and awareness of the self-regulatory bodies and the networks themselves to fill these gaps and guarantee the quality of communication between brands and the public [23,24]. 

### 1.2. The Social Responsibility of the Media and Unfair Advertising

The relationship between media and society has always been problematic in two areas [25]. On the one hand, the media control and influence society through preparation, agenda setting, framing and publicity, according to their commercial and political interests. On the other, the huge changes and the dynamics of information technologies make the standards and regulations of the media become obsolete very rapidly [26,27,28]. At this point, the contradiction that exists when a medium spreads misleading advertising can already be sensed, where the responsibility falls on several of the agents: advertiser, agency and medium [28].

Corporate Social Responsibility is a strategic management system that integrates, applies, develops, verifies and evaluates the behaviour of actions in the respective businesses and institutions [28,29]. CSR bases its theoretical development on business management and social marketing [30]. For Campos [30], in the communication sector, CSR takes on special relevance as “the complexity of communication and modern media companies currently requires a broader, comprehensive, convergent and total approach to this responsibility not only to fulfil its social purpose, but also to regain or not lose the credibility that sustains the essence of mediation”. In the case of media companies, socially responsible management converges [31] with the management of responsible communication [32,33]. 

In general, both in the US and in Europe there is a sensitivity and its consequent regulation of the media in terms of social responsibility. Advertisers are primarily responsible for all commercial communications for the audience [34]. The good practices of social responsibility of a communication medium are evaluated, among other things, by the quality of informative content, by its programming and the good management of advertising [35]. 

If the frequency of complaints about unfair advertising is analysed, it can be verified that all media are subject to complaints [36]. However, it is surprising that despite the cases of misleading advertising found on the radio, especially for health-related products, the truth is that this medium is characterized by its low relative weight in the set of claims filed. The cause seems to be found, among other reasons, in that the radio still does not receive sufficient monitoring by the advertising self-regulation body Autocontrol and its scant proactivity to claim illicit advertising [37].

Misleading advertising constitutes a socially irresponsible action at the most basic and elementary levels of corporate social responsibility [28]. Misleading advertising directly harms consumers, as a group of people on whom the company depends to guarantee its existence and durability. If the levels of corporate social responsibility defined by Grunig [38] are attended to, [39] in the field of public relations, then, it is shown that misleading advertising fails to comply with the most elementary level of public responsibility, as deception of the consumer affects one of the organization’s primary commitments, directly related to its basic functions [28]. In addition, and from the business approach, “misleading advertising implies the breach of several dimensions of corporate responsibility, legal of course, but also and especially ethical responsibility, broader and higher ranked” Carroll [40]. 

If illegal, unfair and misleading advertising is discovered, it is due primarily to the social responsibility of advertisers and advertising agencies, or to intuition and disrespectful behaviour in managing relationships with the public [28]. Misleading advertising directly harms one of the interest groups of a company, its current or potential consumers, reason for which it violates the basic principles of socially responsible management [41,42]. As García Nieto [28] indicates, unfair advertising violates the principles of responsibility of different theorists [43,44]. Acts of unfair advertising, specifically misleading advertising, represent fraud and are classified as an administrative offense, so the advertising company can be sanctioned for it. However, not only is this legal dimension found, but also that of social responsibility, and a higher ranking one, such as ethical responsibility [40]. 

The link between advertising and CSR is manifested in the self-regulation itself, where these bodies provide CSR certificates to advertisers who undergo this self-regulation, to include them in their sustainability reports [45]. 

### 1.3. Misleading Advertising of Health Products

Investment in advertising has fallen since the start of the COVID 19 pandemic. Only the health sector has increased investment in advertising, up to 3% [46]. The health sector is defined as the set of values, norms, institutions and actors that develop activities of production, distribution and consumption of goods and services and whose main or exclusive objectives are to promote the health of individuals or population groups [47,48]. In advanced societies, health includes diverse factors such as the social, work and personal environment, as well as the services that promote and maintain health [49]. 

In countries such as Spain, the concept of a healthy life is increasingly integrated into society. Advertising reminds us of the need to eat well, avoid a sedentary lifestyle and stay young and healthy [37] in all kinds of products, even those that have nothing to do with health. Companies advertise their novel foods in a peculiar way, using health as the axis of their advertising messages [50]. Health has been established as a clear advertising claim, not always with truthful arguments; a false image of health is spread, sometimes with fake news and pseudo-fact [51,52]. In fact, most of the advertising claims concern pieces related to products that promise healthy benefits [37]. Therefore, it is inevitable to talk about the responsibility of advertisers. “Advertisers are morally responsible for strategies that incite a certain type of behaviour” [53]. 

A paradigmatic case of deception in the advertising of supposedly healthy products is that of the so-called “miracle products”. It is common for these advertising campaigns to use messages with arguments or references of sanitary appearance. There is high awareness of misinformation in health related advertising [54]. The target audience for these kinds of advertised products is especially weak and easy to succumb to the promises [55]. As can be seen, the misleading advertising of supposedly healthy products affects especially the most vulnerable people, putting their health at risk [56]. For instance, the favourite medium of brands to address the youth audience is television, a medium in which advertising for children of unhealthy foods and beverages predominates over healthy products [57,58,59].

One aspect of concern in the area of misleading health advertising is the use of famous people or experts to provide testimonials about the advertised product. In spite of the legislation establishing several measures and limitations for the use of this practice, some studies reveal that there are many brands that use testimonials [60]. 

However, it must also be borne in mind that health-related advertising, advertising directly or indirectly linked to the health field can also produce anomalies, promote prejudices, generate loss of meaning, produce anomie [56]. Health-related products find an audience more sensitive to persuasion in the vulnerable public, which they benefit from in order to achieve a better performance of business objectives [56].

Health is one of the most worrying issues in relation to the problem of disinformation [53]. The data provided by various investigations reveal that the majority of complaints from consumers or interest groups refer to products related to health benefits [37]. Of the complaints filed in consumer associations between 2010 and 2015, Food and Health were the most numerous amongst more than 40 sectors, and in those five years, those of Health increased fourfold [36]. All this despite the sanitary prohibitions developed [61,62]. 

The use of public figures is very common in this type of advertising which, although it does not constitute, in itself, misleading advertising, “these types of claims can be manipulative enough to raise a legal (or at least an ethical) debate within advertising strategies” [63], especially with vulnerable audiences, such as children. 

Actions such as these lead consumers to become victims of scams. The presence of influencers or familiar faces in advertising, or the mere fact that they simply use the product, serves as a push and motive for a purchase. It is contradictory that this happens when the journalists’ own code of ethics prohibits these professionals from collaborating in advertising campaigns for reasons of transparency of the medium and the information professional, and thus avoid conflicts of interest [64,65,66].

One of the five sectors with the most claims in Autocontrol is health [67]. This fact can be increased by adapting Spanish legislation to the five Community Directives that concern advertising communication [68]. These cases confirm the conclusions of a previous study [49] according to which misleading radio advertising is found mainly in the categories of food, beverages, beauty and hygiene and health. In addition, the use of testimonies from people of reference, such as health personnel or opinion leaders, in relation to whom the legislation imposes clear restrictions and prohibitions is surprising [60].

In conclusion, misleading advertising is a reality despite the legislation and self-regulation of the sector, not so much because of the tacit intention to deceive, but because of an extreme use of advertising language that plays with the perception and interpretation of the public, either by the questionable use or absence of their elements [69].

### 1.4. Advertising on the Radio

Nowadays, the radio can be considered as much an advertising medium as it is an informative or entertainment one. A medium that, due to its characteristics, stimulates the imagination and consequently encourages creativity [70] and one that has always seen in technology an opportunity to continue its work [71]. However, radio advertising does not always receive the attention and professional dedication it deserves, to the point of making it “repetitive, monotonous, old-fashioned, realistic, unimaginative and exaggerated” [49]. All this, despite the fact that the transmission of advertising messages on the radio is constructed in a more personal and intimate way, making the listener receive the message in a particular way, as if the broadcaster were only addressing him or her [69].

The study of the Advertising Observatory [72] reveals that almost half of the Spanish population, 22,930,000 million people, listen to the radio. The radio occupies the third position in Spain by volume of advertising investment, after digital media and television, with a contract of 374,9 million euros in 2020 [46]. Its consumption, despite the passing of time and the advancement of technology, remains conventional and traditional, with a loyal audience, of which more than half of the listeners do not change stations during advertising [73].

However, not all advertising formats are valued in the same way by the public. Thus, according to the aforementioned study by the Advertising Observatory [73], commercial and sponsorships are the best valued, with a score of 6.4 and 6.3 respectively (on a scale of 1 to 10), while straight announcement and microprograms obtain a score of 4.7 and 4 respectively. 

When advertising investment by sector is analysed, we see how financial institutions continue to be the ones that opt for the radio medium, confirming the trend marked from previous years. Finances represent 17% of radio advertising investment; public and private services, 14.4%; culture, education and the media, 13.4%; health, 11%; and transport, travel and tourism, 9.4% [34]. The radio continues to be a medium of great interest for advertising in these sectors.

The radio is an instrument for education and its potentialities are implicit when it fulfils the functions of educating, informing and guiding society in a dynamic way, with messages for the promotion of health and prevention of diseases [71]. In some more disadvantaged areas, radio is an opportunity for health messages due to its easy access versus that of the television [74].

## 2. Objectives and Methodology

The main objective of this article is to know the perception and opinion that advertising communication professionals have about misleading advertising and their responsibility in relation to it. Specifically, the paper focuses on misleading advertising in relation to health-related products on the radio medium.

Different research questions were posed:

P1 What responsibility do these professionals want to assume and do they assume in the advertising process?

P2 What knowledge do professionals have about laws and liability?

P3 How does it influence the creative process and the use of rhetorical resources?

P4 What media are considered by professionals as the most prone to mislead advertising? Is radio one of them?

In order to achieve this objective, a quantitative study [75] has been carried out through the application of a survey with closed responses [76]. The sample was made up of 68 advertising professionals belonging to the most important agencies in Spain. Both creative and account executive profiles were included in the sample, since both collaborate in the design of the strategy and the message to be transmitted once the briefing of the advertiser is received. The investigation has a descriptive-exploratory nature; this the sample is not representative. The reason lies in the impossibility to determine the number of advertising professionals in Spain, since, despite the numerous associations, there is no individual one that can be said to group the majority. However, those profiles that make direct decisions regarding the content of the message have been surveyed: creatives and account executives (including planners), leaving aside other profiles such as media planners, who do not intervene in the essence of the message.

The questionnaire had 31 questions, dichotomous or multiple-choice answers, and a Likert-type scale, except one with a closed response. The results of this survey will make it possible to analyse who is responsible, the creative process and respect for the law, assessment of responsibility in advertising, knowledge of the laws, assessment of the different media and compliance with responsibility. Even though this sample cannot be considered quantitatively representative, it does have exploratory validity as it reflects the range of diverse opinions of the aforementioned professionals. Therefore, this article is a descriptive exploratory research.

Prior to the dissemination of the survey, a series of in-depth interviews with advertising professionals had been carried out, the results of which were taken into account for the design of the questionnaire. 

## 3. Results

Advertising professionals for advertising companies and agencies work cooperatively to build successful campaigns that position brands in the minds of consumers. However, these professionals may have different views on the same issues due to the different perspectives from which they work. It is interesting to find out what these opinions are and to know the perception of responsibilities in the process of preparing a campaign depending on the side from which it is viewed. 

The questions were divided around two main issues: their experience and professional practice in customer relations and their opinion on the practice of advertising related to health and the media. 

In the first section, regarding the level of demand of advertisers, 72.1% have described their clients as very demanding. On the other hand, 23.5% consider that only some of the clients are, but not the majority. Finally, 4.4% do not consider the clients they work for demanding. When coding the variables on a scale from zero to two in terms of level of demand, the mean is set at 1.68 with a median and mode of 2.

The most common demands of clients are enquired through an open answer. We observe that almost half of the respondents refer to budget-related requirements (41.17%). Secondly, the fulfilment of the briefing requirements (20.58%) and the time to carry out the campaigns (17.64%) stand out. Lastly, highlighting the logo of the advertised brand. 

Only 8.8% of those surveyed have assured that these types of demands are not a determining factor in the creative process. Almost 60% have indicated that it is more difficult to work on a creative campaign that has many conditions, while 32.4% indicate that it is complicated, but they do not consider that it conditions their creativity excessively. Coding from zero to three in terms of the level of demand and its conditioning of creativity allows us to establish a mean of 2.29 with a mode of 2 and a median of 3.

38.2% of those surveyed state that, when it comes to an advertiser that is complicated in the legal sense, due to the brand or the product category, less than half of the briefings collect the necessary indications to develop a campaign. On the other hand, 31% declare that these guides are indicated in all or most of these briefings. When cleansing the variables and coding them on a scale from zero to three, the mean is 1.92 with a mode and median of 2.

Looking at the campaigns from a creative point of view, there is a clear division between advertising professionals. 47.1% of those surveyed state that the creative person in charge of a campaign is the agency, while 50% think that both the agency and the advertiser are responsible. 

**Figure 1:**
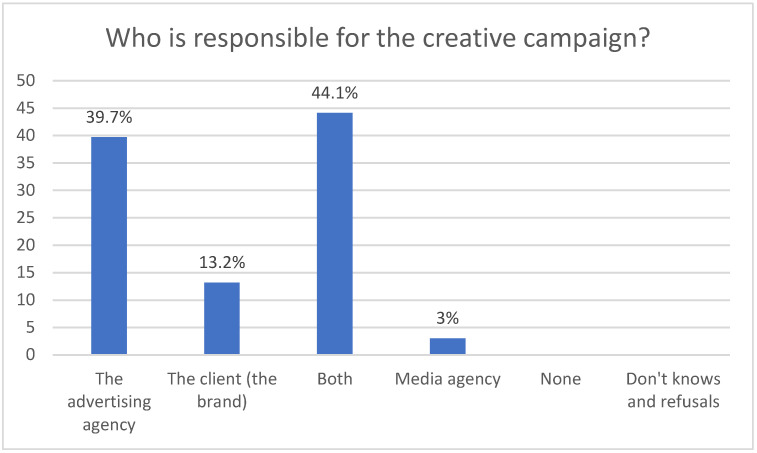
Who is responsible for the creative campaign.

**Figure 2:**
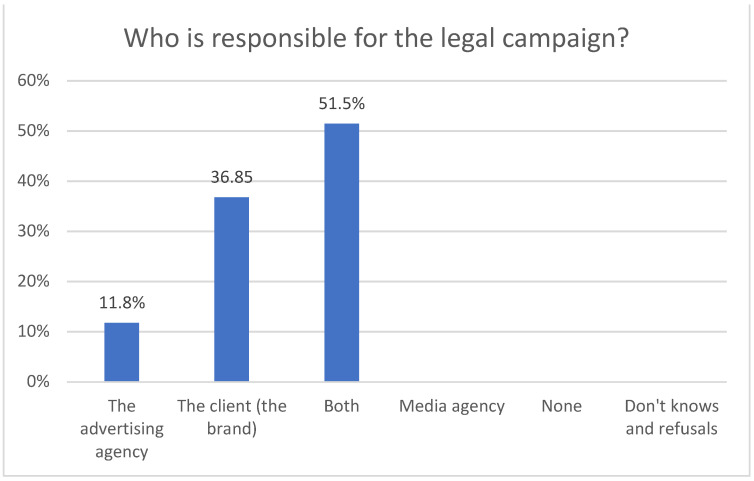
Who is responsible for the legal campaign.

Regarding the legal responsibility in relation to a campaign, 50% of advertising professionals consider that both the client and the agency are responsible, 13.2% consider that it only belongs to the advertising agency and 36.8% attribute the legal responsibility to the advertiser (Figure 1 and Figure 2).

As a result of the above, 83.3% of those surveyed assure that advertising should always be responsible. On the other hand, 11.8% consider that it should be, but it is not always necessary. 

From the answers to the question, with multiple answers, about the meaning of “being responsible” in advertising campaigns, it follows that for 76.5% of those surveyed, being responsible means respecting the values that prevail in society. Secondly, it has to do with compliance with legal requirements (51.5%). Less than half (45.6%) believe that responsibility implies “think about the consumer”, and that takes into account the environment (38.2%). In addition, among the open responses collected, a call is made to “staying away from misleading advertising”; “being honest. Not lying”; “not sending offensive messages”.

After assessing what responsibility means, they were asked if they believe that advertising is responsible. Only 10.3% of the people surveyed consider that advertising is responsible in all its aspects and forms. More than half of those surveyed (57.4%) believe it depends on the product or brand being advertised, and 26.5% associate it with the advertising company. 

Regarding advertising legislation, advertising professionals agree that it is necessary to have knowledge of legislation on the part of the advertiser (85.3%), the media (91.2%) and the advertising agency (88.7 %). 

**Figure 3:**
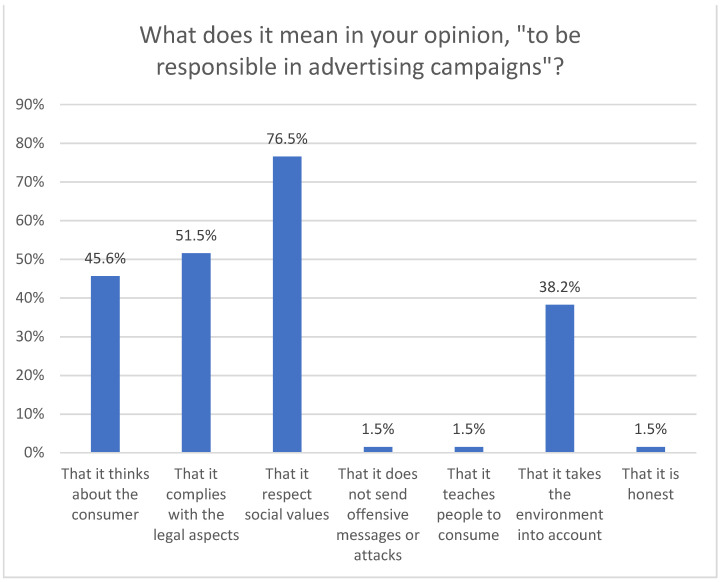
Liability criteria.

For half of the respondents (51.5%) it is important to know the advertising legislation when carrying out their work. In fact, about 20% consider it essential. However, it is surprising that almost 12% answered that knowing the legislation is not at all or not very important. For 37% of those surveyed, knowledge of the law and its application has some importance, but it is not perceived as something significant in the exercise of their profession (Figure 3).

Only 10.3% know in depth the advertising legislation and almost half of those surveyed claim to know it, but not in depth, while 39.7% claim to know only specific aspects of the legal regulations. However, 19% have revealed that they never consult the legislation and half of those surveyed have answered that only on specific occasions. 

Different opinions are observed regarding the barriers determined by the law. 38.2% assure that the legislation establishes barriers in the creation process and is affected by them. 36.8%, however, do not feel affected by them. 25% answer that “maybe”. However, when professionals are asked about the extent to which they consider that the law hinders work on a scale of 1 to 5, with 1 being not at all and 5 being a lot, most of the responses concentrate on the number 2 (36.8 %) and 3 (33.4%), so legal limitations are not considered to excessively hinder the creative process. 

On the subject of advertising deception in relation to the consumer, on a scale of 1 to 5, with 1 being nothing and 5 being a lot, 83.5% consider it very dangerous. In addition, most of the respondents say that it occurs only occasionally, while 25% say that it occurs frequently. 

**Figure 4:**
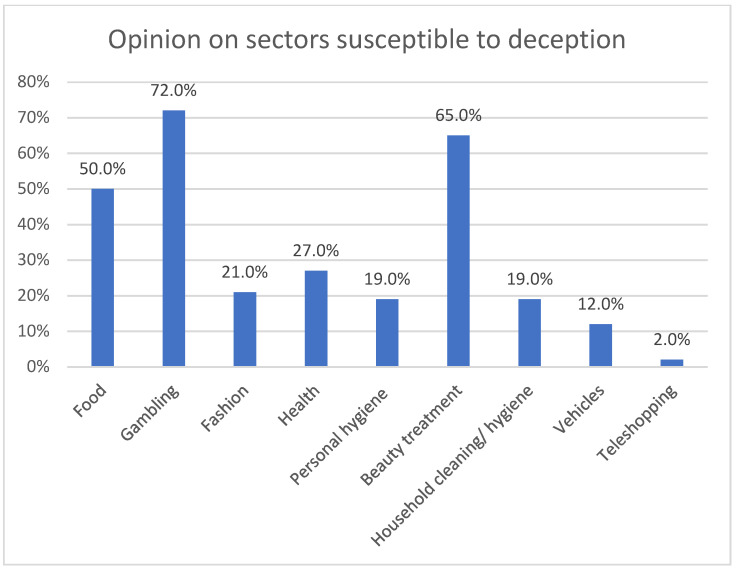
Opinion on sectors susceptible to deception.

When asked about the sectors most susceptible in which deception (Figure 4) is more frequent, the majority of those surveyed associate it with gambling and, after this, with the beauty treatment sector (65%) and the food sector (50%). 

The health sector, which is the object of this investigation, is not mentioned as a sector particularly prone to advertising deception, along with the sectors of fashion, personal hygiene products and household cleaning and hygiene. However, professionals do recognize that it is in the health and food sectors that misleading advertising is most dangerous.

72.05% of those surveyed state that they fully agree with the statement “misleading advertising should be eradicated” and almost 15% have indicated they agree. On the other hand, 69.1% of the professionals have shown to fully agree with the statement “advertising deception should have more control”, while 19% have shown to agree. In addition, 67.4% fully agree that making use of advertising deception directly affects the credibility of a brand. 

A greater division of opinions is observed in the proposal that campaigns, based on recommendations through testimonials from consumers or famous people, should be more regulated. 33.8% totally agree with the statement, 26.4% agree, 25% consider themselves indifferent, 7.35% disagree and the same proportion totally disagree.

On the other hand, advertising professionals consider that the medium in which it is easier and more frequent to induce deception is the internet –indicated by 92.6%–, especially by social networks. This is followed by television (66.2%); and with a visibly lower percentage, in third place, magazines and radio are pointed out, both with 13.2%. 

**Figure 5:**
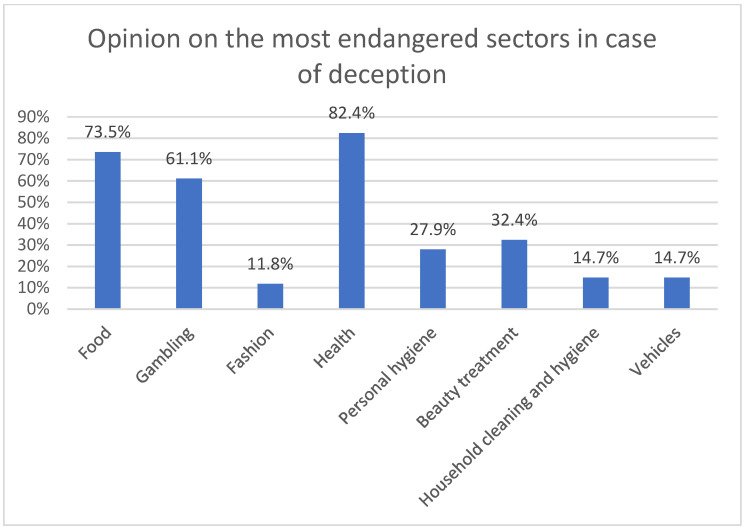
Opinion on the most endangered sectors in case of deception.

If we relate variables, we find issues to consider. Even though the health sector is not perceived as one of the three most susceptible to deception, it is considered the most dangerous if it occurs. The opposite case is that of the Beauty treatment sector, which leads to consider whether the sector considers that the legislation protects the Health sector more than that of the Beauty treatment one (Figure 5).

**Figure 6:**
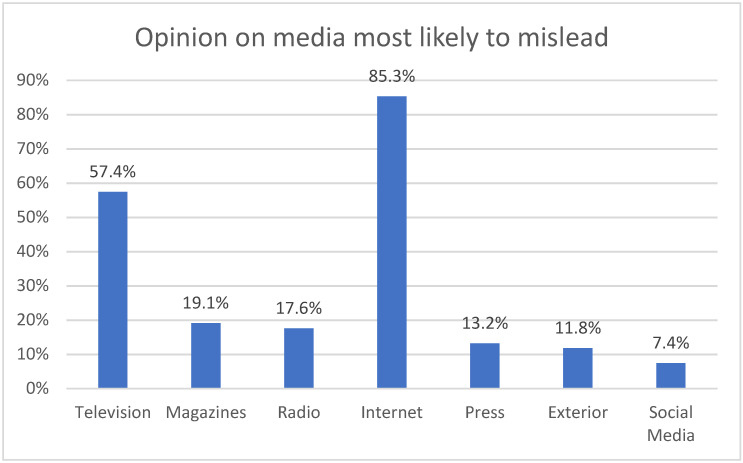
Opinion on media most likely to mislead.

This question establishes a clear relationship between the methodology and Q4 of the article, since it focuses on misleading advertising in relation to health-related products in the radio medium (Figure 6). Specifically, in the health sector, 88.2% of those surveyed consider the internet as the medium where more deception can occur, followed by television (55.9%). In addition, magazines (19.1%), the press (16.2%), radio (14.7%) and outdoor advertising (13.2%).

Finally, 88.2% of those surveyed consider it favorable that the health sector has more legislative restrictions than the rest of the sectors, including the food sector. 

## 4. Discussion 

It is of great interest to know the opinion of the professionals who prepare advertising messages related to health, to sell these products to all types of consumers. Professionals argue that their job is to create persuasive content that helps their clients, advertisers, meet a goal. Likewise, they understand that the creative process carried out in the agencies is undoubtedly and, in any case, a task of responsibility with the public due to the impact it has on society. The balance between persuasion and consumer protection, together with the collaboration between agency and advertiser is the key. 

The advertising process has gaps in the control of misleading advertising. Advertising agency professionals work for numerous advertisers normally belonging to different sectors, amongst which are those that are subject to the strictest legal regulations in relation to their advertising campaigns [77].

However, after having collected the opinions and experience of professionals, only 10.3% claim to know the law in depth. Most advertisers confirm that they have legislative knowledge but admit that it is not in depth and only about some specific issues. In addition, 4.4% of those surveyed even confirm that they completely lack this type of knowledge. 

Agency professionals feel safe in this regard because many of the briefings include clauses on legal limits. In addition, many of the large advertising agencies have a legal service that controls the progress of creative pieces so as not to commit fraudulent acts. However, almost 90% of those surveyed consider it necessary to have legislative knowledge, but for only half is it important. It is striking that, for 12% of those surveyed, knowing the legislation is not at all or not very important. For this group of advertisers, knowledge of the law is irrelevant in the performance of their work. Even for 37% the legal question is not considered significant in their profession. 

Shared assumption of responsibility for the creation process. 

If we refer to the responsibility of the campaign creation process, we find differing opinions among the professionals of the advertising agencies. For half of those surveyed, the responsibility for the creative process of a campaign corresponds to both the client and the agency. For the other half, however, it is only the advertising agency that is responsible for its creation.

From a legal point of view, half of those surveyed believe that responsibility for the campaign also rests with both the agency and the advertiser, while another 36% attribute legal responsibility solely to the advertiser. A further 13.2% attribute this responsibility only to the advertising agency. 

Health-related products are considered the most dangerous in the case of misleading advertising. 

Even though the work of advertising professionals within agencies covers many sectors due to the number of brands they work with, they have agreed that there are sectors more dangerous than others when misleading. It has been possible to observe the conformity of advertising professionals with respect to the health sector, mentioned by the vast majority as one of the most vulnerable and dangerous sectors in relation to misleading advertising. In addition, it is ensured that this should be one of the most protected sectors within the advertising world and, it should be noted, that the vast majority of the professionals surveyed see the legal restrictions on advertising in the health sector as positive. 

The radio is valued as a serious medium where misleading advertising is unlikely. 

The professionals of the agencies do not consider the radio as one of the main mediums where publicity deception can be induced in the health sector, this is the object of study. In fact, radio is behind other media such as the internet, television or magazines, when it comes to advertising deception in general. This is also the case if you ask about the health sector in particular. Radio ranks fourth behind the aforementioned media. Interestingly, advertising professionals do not consider deception to occur on the radio. In fact, radio is described as a serious medium in which the broadcasting of deceptive campaigns is considered almost impossible and highly unlikely.

## 5. Conclusions

To conclude, the fact that agency professionals do not perceive the existence of misleading advertising in the health sector may be subject to study if we compare it with previous studies [14,19]. They do not consider radio as one of the media where this deception can occur the most, however, most of the advertising claims refer to pieces related to products that promise health benefits [37]. Even though, they agree that the health sector is one of the most dangerous if one takes into account the damage that advertising deception can cause to consumers, who in many cases are vulnerable [56]. 

## Data Availability

Data sharing not applicable.
